# Insights into Basal Signaling Regulation, Oligomerization, and Structural Organization of the Human G-Protein Coupled Receptor 83

**DOI:** 10.1371/journal.pone.0168260

**Published:** 2016-12-09

**Authors:** Anne Müller, Julia Catherine Berkmann, Patrick Scheerer, Heike Biebermann, Gunnar Kleinau

**Affiliations:** 1 Institute of Experimental Pediatric Endocrinology, Charité-Universitätsmedizin Berlin, 13353 Berlin, Germany; 2 Institut für Medizinische Physik und Biophysik, Group Protein X-ray Crystallography and Signal Transduction, Charité-Universitätsmedizin Berlin, Berlin, Germany; University of Michigan, UNITED STATES

## Abstract

The murine G-protein coupled receptor 83 (mGPR83) is expressed in the hypothalamus and was previously suggested to be involved in the regulation of metabolism. The neuropeptide PEN has been recently identified as a potent GPR83 ligand. Moreover, GPR83 constitutes functionally relevant hetero-oligomers with other G-protein coupled receptors (GPCR) such as the ghrelin receptor (GHSR) or GPR171. Previous deletion studies also revealed that the long N-terminal extracellular receptor domain (eNDo) of mGPR83 may act as an intra-molecular ligand, which participates in the regulation of basal signaling activity, which is a key feature of GPCR function. Here, we investigated particular amino acids at the eNDo of human GPR83 (hGPR83) by side-directed mutagenesis to identify determinants of the internal ligand. These studies were accompanied by structure homology modeling to combine functional insights with structural information. The capacity for hetero-oligomer formation of hGPR83 with diverse family A GPCRs such as the melanocortin-4 receptor (MC4R) was also investigated, with a specific emphasis on the impact of the eNDo on oligomerization and basal signaling properties. Finally, we demonstrate that hGPR83 exhibits an unusual basal signaling for different effectors, which also supports signaling promiscuity. hGPR83 interacts with a variety of hypothalamic GPCRs such as the MC4R or GHSR. These interactions are not dependent on the ectodomain and most likely occur at interfaces constituted in the transmembrane regions. Moreover, several amino acids at the transition between the eNDo and transmembrane helix 1 were identified, where mutations lead also to biased basal signaling modulation.

## Introduction

The G-protein coupled receptor 83 (GPR83 [1]) is a single copy gene and was first described as glucocorticoid-induced receptor (GIR; also termed GPR72 and GPR73). Gpr83 is most abundantly expressed in the murine brain and the thymus [2, 3] and was found to play a regulatory role in thermogenesis and to participate in the control of circulating adiponectin levels [4]. The *Gpr83* knock-out mouse is protected against diet-induced obesity and mGPR83 has been suggested to be a determinant in systemic energy metabolism [5]. Several different G-protein coupled receptors (GPCRs) have been identified to be involved in metabolism and body weight regulation such as the melanocortin-4 receptor (MC4R) or the ghrelin receptor (GHSR) (reviews [6, 7]).

Moreover, recently published experiments have provided first insights into molecular and pharmacological properties of GPR83. Briefly, the C-terminal fragment of neuropeptide Y (NPY) can bind to GPR83, but with a significantly lower affinity compared with NPY receptor subtypes [8]. However, NPY receptor binding has not been shown to induce signaling at GPR83. In contrast, the neuropeptide PEN was recently described as a potent GPR83 ligand [9]. In addition, murine GPR83 basal inositolphosphate (IP) formation is increased by treatment with zink ions (Zn^2+^) or/and several constitutively Gq activating mutations have been designed and characterized [10], including an N-terminal deletion variant, which suggested a potential role of the N-terminus as an intra-molecular antagonist [11]. This potential intra-molecular ligand can be defined more generally as a *tethered ligand*. In addition, homo- and hetero-oligomerization with the ghrelin-receptor (GHSR) was described for mGPR83 and this interaction diminishes GHSR activation by acyl-ghrelin [5]. Hetero-oligomerization and a mutual functional impact of GPR83 with GPR171, a receptor for the peptide bigLEN, have also recently been shown [9].

In summary, the molecular properties and regulation of GPR83 are complex, including: *i*., a potential tethered ligand action [11, 12]; *ii*., exhibition of basal signaling [10]; *iii*., a multi-facet signaling capacity by stimulation with PEN (e.g., Gq and Gi); and *iv*., the capacity to homo- and hetero-oligomerize with various functional impact [5, 9]. Of note, constitutive (ligand independent) signaling is known as a regulatory element of GPCRs, which has dramatic concomitant consequences for receptor reactivity concerning ligand binding and the signaling spectrum [13–17]. Endogenous basal signaling is related to the specific physiological role of the particular GPCR, e.g., for low energy consuming maintenance of constant signal induction linked with a specific biological cell or tissue function.

The motivation of this current study was to investigate particularly detailed extracellular amino acids of hGPR83 that contribute to the regulation of basal signaling and may be part of an intra-molecular ligand. Secondly, the influence of hetero-oligomerization on basal signaling properties of interacting GPCRs was analyzed. For this purpose, we designed and tested hGPR83 variants, including an N-terminal deletion construct and single or multiple amino acid substitutions at highly conserved extracellular regions. These studies were accompanied by modeling studies, either of GPR83 alone or in specific complexes to estimate a more comprehensive understanding of the functional data, which are ultimately related to the 3-dimensional properties of GPR83.

## Material and Methods

### Vector construction

*Gpr83* was amplified from murine hypothalamic cDNA [10]. *GPR83* was amplified from human hypothalamic cDNA. *5HTR1B*, *MC3R*, and *MC4R* were amplified from human genomic DNA and GHSR from human cDNA (UMR cDNA Resource Center, Rolla, MO, USA). The pcDps [NHA-rM3R], origin of the untagged rM3R construct (rat cholinergic receptor, muscarinic 3), was kindly provided by Torsten Schöneberg (University of Leipzig, Germany). Receptor cDNAs were cloned into the pcDps expression vector. The hemagglutinin (HA) tag was inserted at the amino terminus or downstream of the signal peptide. The Flag tag was generated at the carboxyl terminus. The origin of YFP fragment constructs were pcDNA3 [*MCFD2-YFP1/YFP2*] plasmids, which were kindly provided by Hans-Peter Hauri (University of Basel, Switzerland) [18]. For interaction analysis of two known proteins in YFP-BiFc, *MCFD2* was replaced by the here relevant genes (without stop codons). The deletion mutant hGPR83-del17-60 was created in two PCR steps. The single amino acid mutants of hGPR83 were created by site-directed mutagenesis. Automatic sequencing was used to determine the accuracy of all plasmids. The pGL4.30[luc2P/NFAT-RE/Hygro], pGL4.33[luc2P/SRE/Hygro], and pGL4.34[luc2P/SRF-RE/Hygro] reporter constructs, co-transfected for analysis of IP3 formation, ERK1/2 and RhoA activation were purchased from Promega (Mannheim, Germany).

### Cell culture and transfection

COS-7 cells were cultured in Dulbecco´s modified medium (DMEM/Biochrom, Berlin, Germany) and HEK293 cells were cultured in minimal Essential medium (MEM/Biochrom, Berlin, Germany). Both cell lines were supplemented with 10% fetal bovine serum, 100 U/ml penicillin, and 100 μg/ml streptomycin and incubated at 37°C in a humidified 7% CO2 incubator.

For cell surface expression studies, COS-7 cells were seeded into 48-well plates (3.8 x 10^5^ cells/well) and transfected with 167 ng DNA and 1 μl MetafecteneTM/well (Biontex, Martinsried, Germany). For cAMP accumulation, COS-7 cells were seeded into 96-well plates (0.9 x 10^4^ cells/well). For measurement of NFAT, SRE, and SRF activity via reporter gene assays, HEK293 cells were seeded in 96-well plates (1.5 x 10^4^ cells/well) coated with poly-L-lysine (Biochrom). Transfection in 96-well plates was performed with 41.7 ng plasmid DNA/well and 0.5 μl MetafecteneTM/well. For reporter gene assays, equal amounts of the appropriate reporter construct containing the firefly luciferase gene were co-transfected. For bimolecular fluorescence complementation (BiFc) approaches, HEK293 cells were seeded in 6 cm dishes (8.5 x 10^5^ cells/dish) and transfected with 1.9 μg DNA: 5.9 μl MetafecteneTM/dish. Interaction studies via sandwich ELISA were performed with COS-7 cells seeded in 6 cm dishes (6.5 x 10^5^ cells/dish) and transfected with 3 μg DNA and 8 μl MetafecteneTM/dish. All transfections were performed one day after seeding.

### Cell surface expression studies

Cell surface expression studies of wild type hGPR83 and designed constructs were carried out in COS-7 cells and performed using an ELISA system that detects HA-tagged receptors. Cell surface expression analysis was undertaken 48 h after transfection. A tag-less hGPR83 served as a negative control. For detection of the HA-tag, cells were washed, fixed with paraformaldehyde, and probed with a biotin-labeled anti-HA antibody (Roche Applied Science, Mannheim, Germany). Bound biotin anti-HA antibody was detected by peroxidase-labeled streptavidin (BioLegend, London, UK) in a substrate/chromogen reaction as previously described [19].

### Measurement of signaling pathways

Signaling pathways were analyzed 48 h after transfection. Intracellular cAMP accumulation for the determination of Gs/Gi activation was analyzed using the AlphaLISA technology (PerkinElmer, Rodgau, Germany). Human α-MSH was purchased from Sigma-Aldrich (Taufkirchen, Germany). Forskolin was used to investigate Gi activity. Stimulation was performed for 45 min. Cell lysis (50 μL/well lysis buffer) and cAMP measurement was conducted as described elsewhere [10] and according to the manufacturer´s protocol (PerkinElmer).

NFAT, SRE, and SRF activity were determined in luciferase reporter gene assays. Cell lysis was performed with 50 μl/well of 1x passive lysis Buffer (Promega). Pathway activities were determined by luciferase activity according to the manufacturer’s protocol (Promega).

### GPR83 homo- and hetero-oligomerization

Interaction was measured 48 h after transfection using two independent approaches. For sandwich ELISA studies, HA- and Flag-tagged constructs were co-transfected. Cell lysates were generated as described elsewhere [10] and incubated in anti-Flag antibody (Sigma-Aldrich)-coated 96-well plates for 2 hours. The HA epitope was detected after washing with a biotin-labeled anti-HA antibody (Roche Applied Science, Mannheim, Germany). The bound biotin anti-HA-antibody was subsequently detected by peroxidase-labeled streptavidin (BioLegend, London, UK) in a substrate/chromogen reaction as previously described [19]. Total protein concentration of lysates was measured using a bicinchoninic acid (BCA) based protein assay (Thermo Scientific).

For YFP-BiFC, -YFP1, and -YFP2 constructs were co-transfected. Interaction between -YFP1 and -YFP2 constructs reconstitute the active YFP [5, 18]. YFP-fluorescent cells were measured using the FACS CantoII at the *Berlin Brandenburg Center for Regenerative Therapies* (BCRT, Charité Berlin) and were analyzed with FlowJo 8.8.6 (Tree Star Inc., Ashland, OR, USA).

### Statistical analyses

All data was examined for normal distribution and expressed as mean ± or + standard error of the mean (SEM). Statistical analyses were performed using the statistical tools implemented in Graph Pad Prism, version 6 (GraphPad Software, San Diego, CA, USA).

### Structural homology models of GPR83 in different activity states and in interaction with intracellular effectors

No direct structural information is yet available for the GPR83. However, a large number of GPCR crystal structures in different activity state-related conformations have been published in recent years [20]. These complex structures serve as templates for family A GPCR homology modeling with the purpose of studying details of ligand binding or signal transduction. In the present study, we built several different GPR83 homology models, also as a complex with intracellular binding partners. Unfortunately, these models show several limitations such as lack of an entire ectodomain, as no structural template is yet available. Moreover, the putative binding mode of PEN, a GPR83 ligand [9], cannot be included in complex models, because of missing structural information of both the eNDo and PEN, or concrete data on ligand binding determinants.

All structural modifications to generate homology models were performed using the software Sybyl X2.0 (Certara, NJ, US). The AMBER F99 force field was used for energy minimization and dynamic simulations.

#### a. An inactive hGPR83 model based on rhodopsin

For structural modeling of the inactive state conformation of hGPR83, crystallized rhodopsin was used as an initial template (PDB entry 1F88 [21]). In addition to usual modifications for template preparations such as removal of the ligand or loop length adjustments [22], further modifications were performed including substitution of the entire extracellular loop (ECL) 2 by an alternative template. The ECL2 is located close to the transmembrane helices in rhodopsin and opsin, almost parallel to the membrane and tightly closing the binding pocket of the permanently bound ligand retinal. In available crystal structures of peptide receptors (such as the GPR83), this spatial localization differs, whereby the fold of the ECL2 is similar that of the rhodopsin ECL2. In the neurotensin (NTSR1, PDB entries 4GRV, 4XEE) and the angiotensin 1 receptors (AT1R, PDB entry 4ZUD) [23–25], the spatial ECL2 localization is oriented more towards the extracellular space compared with the rhodopsin/opsin ECL2. Similar properties for the ECL2 can be observed at the nociceptin/orphanin FQ receptor (NOP or ORL-1, PDB entry 4EA3, [26]) or in the delta-opioid receptor (DOR, PDB entry 4EJ4, [27]). Therefore, we substituted the ECL2 of the active-like neurotensin receptor (PDB entry 4XEE, [23]) into our rhodopsin template. Moreover, amino acids of the N-terminal part of the crystallized neurotensin receptor (i.e., in interaction with the ECL2) were also substituted into the structural template to receive a fragmental template structure for amino acids that were tested in our hGPR83 mutagenesis approach. The hGPR83 model includes residues Phe55-Asn67 of the eNDo.

Amino acids of this chimeric receptor template were than substituted with residues of the human hGPR83. This was followed by side chain energy minimization until converging at a termination gradient of 0.2 kcal/mol*Å with constraint backbone atoms of the transmembrane helices, which were finally released in a second minimization step until converging at a termination gradient of 0.1 kcal/mol*Å. The entire hGPR83 model is constituted by amino acids from positions Phe55 to Cys363. This first preliminary model was further refined by short molecular dynamic simulations (300 K, 2 ns) and energetic minimizations of the side chain orientations with constrained backbone atoms of helical parts and the beta-sheet like fold of the ECL2 (until converging at a termination gradient of 0.1 kcal/mol*Å). Finally, all constraints were released and the model minimized until a termination gradient of 0.1 kcal/mol*Å.

#### b. An active hGPR83 conformation bound with beta-arrestin

The β-adrenergic 2 receptor (ADRB2) crystal structure in an active conformation (PDB entry 3SN6, [28]) was used as a primary template for modeling the human hGPR83 complexed with a G-protein molecule, which was based on two facts. First, the entire amino acid sequence similarity between ADRB2 and hGPR83 is appropriate for modeling procedures [sequence similarity (31.8%, Blossum62 matrix)]). Second, this active state ADRB2 structure was solved in complex with the G-protein. As described above for the inactive state model, we substituted the ECL2 of the neurotensin receptor (PDB entry 4XEE, [23]) into our major template. The G-protein was defined as a static set and was excluded initially from dynamic simulations. However, the side chains of the entire complex were minimized without any constraint.

#### c. An active state hGPR83 conformation bound with G-protein

The β-adrenergic 2 receptor (ADRB2) crystal structure in an active conformation (PDB entry 3SN6, [28]) was used as a primary template for modeling the hGPR83 complexed with a G-protein molecule, which was based on two facts. First, the entire amino acid sequence similarity between ADRB2 and GPR83 is appropriate for modeling procedures [sequence similarity (31.8%, Blossum62 matrix)]). Second, this active state ADRB2 structure was solved in complex with the G-protein. As described above for the inactive state model, we substituted the ECL2 of the neurotensin receptor (PDB entry 4XEE, [23]) into our major template. The G-protein was defined as a static set and was excluded initially from dynamic simulations. However, the side chains of the entire complex were minimized without any constraint.

#### d. Homodimer and hetero-oligomer models of MC4R and GPR83 based on crystallized GPCR dimer templates

Interfaces between the protomers of GPCR dimers or oligomers were suggested based on experimental data for different GPCRs at the region from TMH3-ICL2- TMH4 [29–31], TMH5-TMH6 [32–34], or TMH4-TMH5 [35]. Furthermore, several crystal structures of dimeric GPCR complexes were also previously determined, such as for the chemokine receptor CXCR4 [36], μ-opioid-receptor (MOR [37]), κ-opioid receptor (KOR [38]), opsin [39], or the β-adrenergic receptor 1 (β-1AR [40]).

In the case of opsin, KOR, and β-1AR, the protomer interfaces are located between TMH1-TMH2 and helix 8, respectively. However, dimer interfaces can be also observed between TMH5-6, e.g., in the crystal structure of the CXCR4. In consequence, in oligomeric states different interfaces could be assumed, which may also be switched during an inter-conversion between monomers and dimers/oligomers [41].

These dimeric or oligomeric GPCR crystal structures are useful to serve as structural templates for the arrangement of family A GPCRs in oligomeric homology models (reviewed in [42]). For modeling of homodimeric GPR83, we used the KOR arrangement and interface at TMH1-TMH2-helix8 (PDB entry 4DJH [38]). By superimposition of the monomeric GPR83 (active conformation from the hGPR83/arrestin complex) with the protomers of KOR dimers, we received an initial hGPR83 dimer arrangement. By fixing the backbone of hGPR83, this general protomer adjustment was subjected to molecular dynamics (2 ns) of the side chains. Short dynamics (0.5 ns) and minimization steps were repeated twice, followed by a final minimization without any constraints.

The same principal procedure as described for hGPR83 was repeated for a MC4R monomer (rhodopsin template provided by the determined rhodopsin/arrestin complex (PDB entry 5DGY, [43]) and dimer homology model. Of note, in difference to hGPR83, the ECL2 of MC4R is constituted by only four amino acids. Therefore, the ECL2 of the template was completely deleted and the four residues were manually added. The model refinement procedure was similar to the above described protocol for the inactive hGPR83 model. This monomeric MC4R was superimposed with the CXCR4 dimer, which shows a protomer interface including the ICL2 (PDB entry 3ODU). For the MC4R, ICL2 is known to be involved in the formation of MC4R homodimers [44–46].

Moreover, for the first time, the MC4R has been demonstrated here to heterodimerize with hGPR83. Furthermore, a dimeric heteromer model for hGPR83/MC4R based on superimposition with the protomers of the KOR dimer was built, as described for the hGPR83 homodimer. In consequence, this dimer arrangement is characterized by main contacts in the transmembrane region with a protomer interface at helix 8, TMH1-TMH2, respectively. For such a putative heterodimer arrangement, we also used the monomeric active receptor conformations in consideration that both receptors MC4R and hGPR83 are known to be basally active in signaling. Finally, we exemplarily suggest a hypothetical MC4R and hGPR83 hetero-oligomer complex constituted by homodimers. In this complex, the homodimer contacts (described above) are maintained, but the interface between both receptor moieties is different compared with the individual interfaces. In case of this suggested hetero-oligomer arrangement, the interface would be located between TMH6-7 (MC4R) and TMH4-ICL2 (GPR83). In conclusion, the hetero-oligomer interface should be asymmetric and different to the homo-oligomer interfaces.

## Results

### Increased constitutive signaling pathways by deletion of the hGPR83 ectodomain

**Fig 1:**
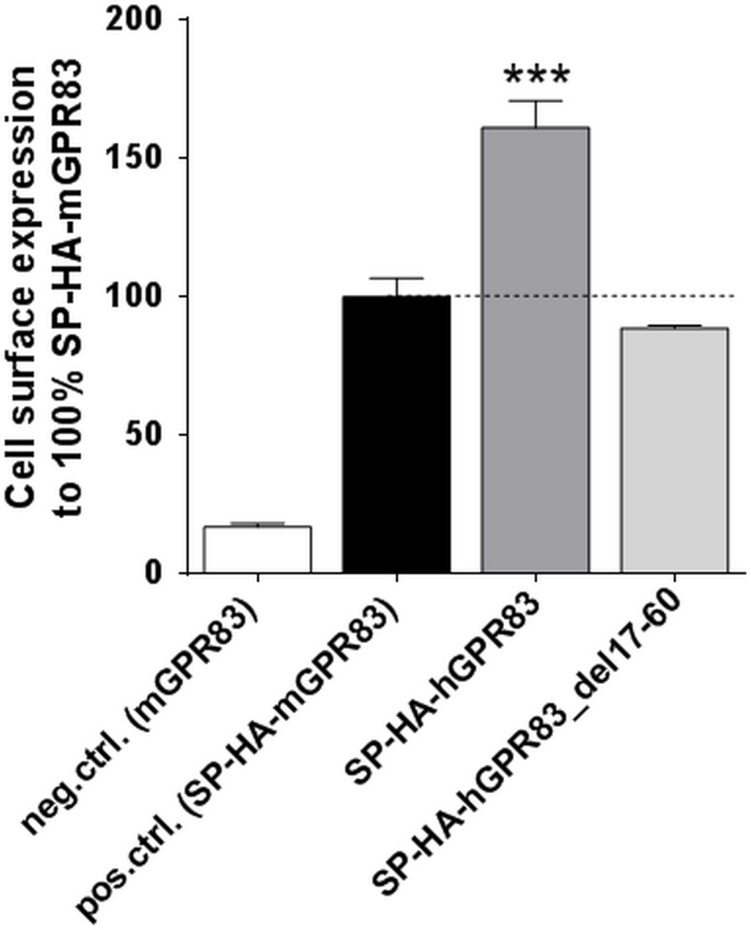
Cell surface expression levels of murine mGPR83, human hGPR83, and an N-terminal hGPR83 deletion construct. Cell surface expression levels of mGPR83 and hGPR83 expressed in COS-7 cells were examined using an ELISA system detecting the HA-tag. Untagged wt mGPR83 was used as a negative control. Data was assessed from a minimum of three independent experiments, each performed in triplicate, and calculated to 100% of the positive control. Values represent mean + SEM. Significance was determined compared with the positive control. ***P ≤ 0.001 (one-way ANOVA, Dunnett´s test).

Most previous molecular insights on GPR83 were obtained using the mouse GPR83 for *in vitro* studies [10, 11] or knock-out mice systems [5]. In the present study, we shifted our focus towards the hGPR83 receptor and revealed that wild-type (wt) hGPR83 is highly expressed compared with the murine GPR83 orthologue (Fig 1). Moreover, a designed N-terminal GPR83 deletion construct (SP-HA-hGPR83_del17-60) from amino acid position 17 (after the signal peptide) to position 60 (close to transmembrane helix 1), which comprised of approximately the entire extracellular N-terminal domain, showed comparable cell surface expression as the native murine mGPR83 (SP-HA-mGPR83) and is decreased compared to wt hGPR83 around 40% (Fig 1).

**Fig 2:**
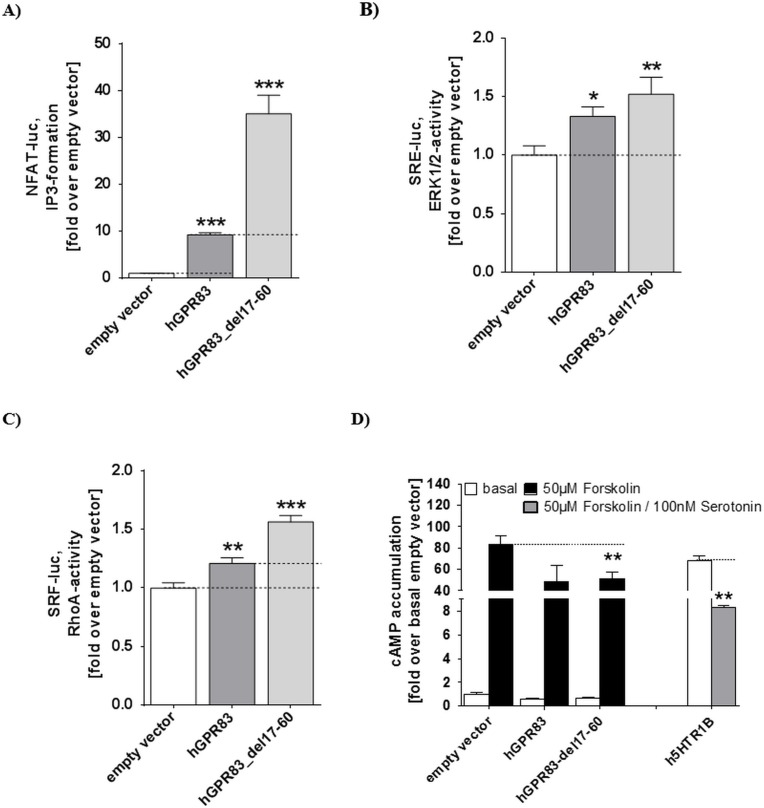
Basal signaling activities of the wild-type hGPR83 and the deletion mutant hGPR83_del17-60. **A)** NFAT-driven luciferase activity (IP3 formation); **B)** SRE-driven luciferase activity (ERK1/2-activity); **C)** SRF-driven luciferase activity (RhoA-activity), and **D)** cAMP accumulation basal and during forskolin stimulation. The forskolin/serotonin co-stimulated serotonin receptor (h5HTR1B) serves as Gi positive control. Data was assessed from a minimum of three independent experiments, each performed in triplicate, and calculated to the basal empty vector set to 1. Values represent mean + SEM. Significance was determined compared with an empty vector using one-way ANOVA and Dunnett´s test (A/B/C and D). Comparisons between wt and the deletion mutant were performed using the unpaired t-test, two-tailed (A and C). ***P ≤ 0.001, **P ≤ 0.01, *P ≤ 0.05 (one-way ANOVA, Dunnett´s test).

This construct exhibits increased constitutive IP formation approximately 4-fold higher than that of wt hGPR83 (Fig 2A). This finding is comparable to previously published data for the mGPR83, which lead to the prior suggestion of an intra-molecular function of this part as a tethered inverse agonist [11]. In addition, the basal signaling capacities for human wt hGPR83 and eNDo deletion variants were compared with regards to specific MAPK, G12/13, and Gi/o activation. The wt hGPR83 shows a slight basal MAPK signaling activity (approximately 1.3-fold), whereby the truncated variant is increased in this pathway by approximately 1.5-fold compared with the empty vector (Fig 2B). In the G12/13 pathway assay, the wt exhibited a slightly increased basal signaling (1.2-fold) and the deletion GPR83 mutant showed enhanced signaling of 1.5-fold compared with the empty vector (Fig 2C). In the Gi-mediated pathway say, a constitutive activity was measured for wt and the deletion variant by a 40% reduction of forskolin-induced cAMP production (Fig 2D), which was not further increased by the deletion mutant.

### Single side-chain substitutions at specific extracellular positions cause ligand-independent basal activities for different pathways

**Fig 3:**
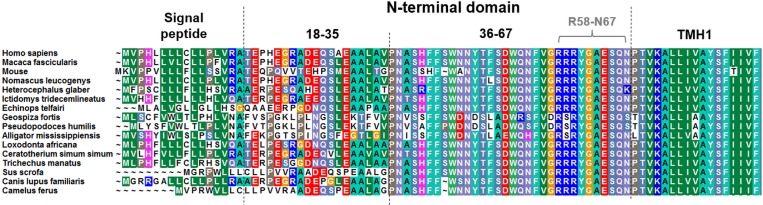
N-terminal sequence alignment of different GPR83 orthologues. The sequence alignment shows that the second half of the N-terminal tail (between positions 36–67) is particularly highly conserved among the compared variants. Therefore, the GPR83 eNDo was subdivided into two regions: 1. a low-conserved region (part 1, positions 18–35) after the signal peptide, and 2. the highly conserved region (part 2, positions 36–67). Without 3D data from structural determination of hGPR83 by crystallographic studies, the N-terminus of TMH1 is only predictable based on comparison with sequences of other family A GPCRs, where crystal structures are available (e.g., beta-adrenergic 2 receptor and rhodopsin). Different colors of amino acids indicate conservation of corresponding positions among subspecies and additionally support discrimination between their biophysical properties: green, hydrophobic; blue, positively charged; and red, negatively charged. The alignment was visualized using BioEdit.

**Fig 4:**
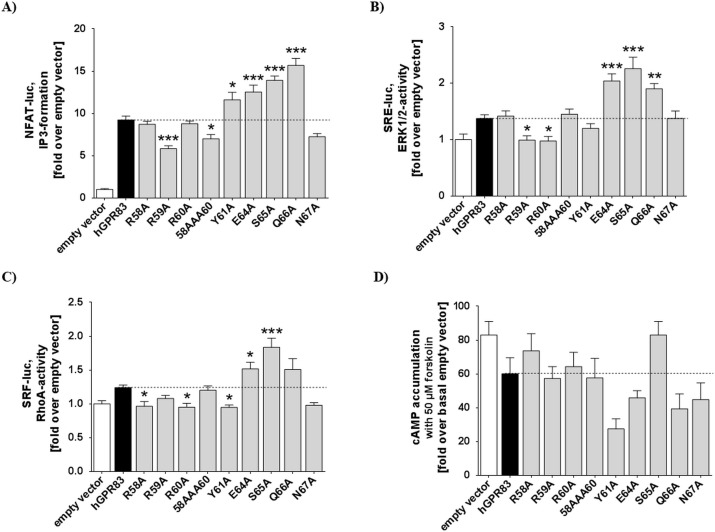
Functional characterization of single side chain variations in the N-terminus of hGPR83. A) NFAT-driven luciferase activity (IP3-formation); B) SRE-driven luciferase activity (ERK1/2-activity); C) SRF-driven luciferase activity (RhoA-activity): and D) cAMP accumulation during forskolin stimulation. Data was assessed from a minimum of three independent experiments, each performed in triplicate, and calculated to the basal empty vector set to 1. Values represent mean + SEM. Significance was determined compared with the empty vector using one-way ANOVA and Dunnett´s test. ***P ≤ 0.001, *P ≤ 0.05.

Of further interest was which amino acids are of functional relevance for the regulation of basal/constitutive activity in the extreme N-terminus of hGPR83. The amino acids at the transition between the eNDo and transmembrane helix 1 (TMH1) are highly conserved among GPR83 orthologues (Fig 3), which indicates a potential relevance for the structural-fold or signaling regulation. Moreover, amino acids at the region between the ectodomain and the TMH1 are known, from other GPCRs, to be of importance for signaling regulation [12]. Therefore, we constructed single amino acid substitutions (to alanine mutants) of these residues (position R58–N67; Figs 3 and 4). Furthermore, a triple alanine substitution of the arginine 58RRR60 motif was designed to evaluate a potential impact of these conserved positive charged residues in a combinatorial way.

The cell surface expression of all hGPR83 mutants was similar to wt (S1 Fig). We also explored the functional properties of receptor variants with regard to Gq and MAPK (Fig 4A and 4B) and also RhoA or Gs/Gi/o signaling (Fig 4C and 4D). These functional studies revealed that the hGPR83 exhibits a high basal Gq/11 signaling activity (8-fold over pcDps (vector alone, negative control)) (Fig 4A). A low but significant increase in constitutive IP3 formation was measured for mutants Y61A-Q66A (around 1.2–1.5-fold). In contrast, the alanine mutants R59A and N67A slightly decreased basal activity to 80% of wt. In the basal MAPK signaling, the substitutions E64A, S65A, and Q66A induced an increase of basal signaling by approximately 40%. The substitutions R59A and R60A reduced ERK basal activity down to pcDps levels (Fig 4B). The RhoA pathway was increased in tendency of the E64A and the S65A mutants (Fig 4C). Of note, the Y61A substitution inhibited cAMP formation by approximately 60%, which indicated constitutive activation of the Gi/o pathway (Fig 4D).

### Impact of the hGPR83 ectodomain on the capacity for homo- and hetero-oligomerization

**Fig 5:**
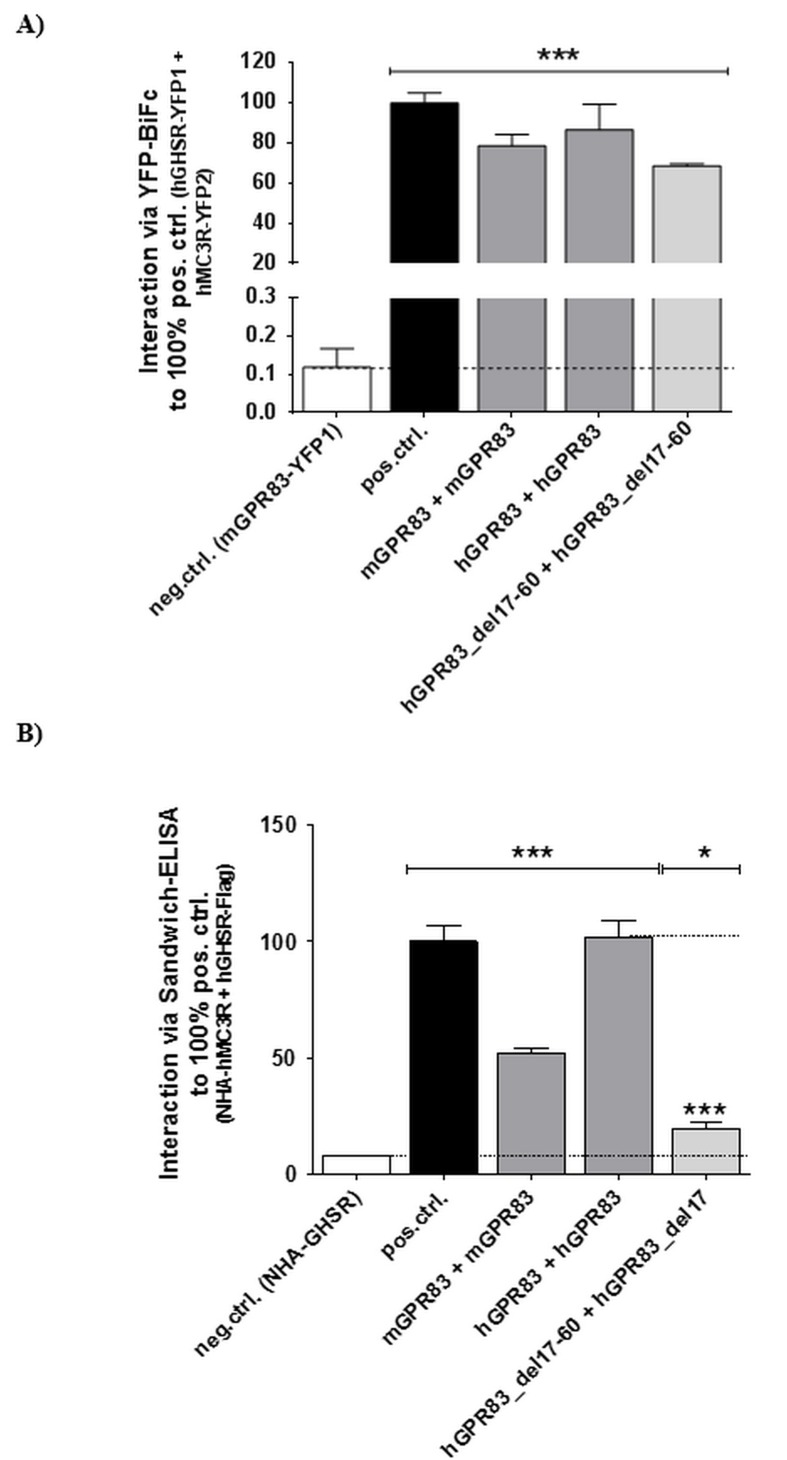
Capacity of wild-type hGPR83 and deletion variant hGPR83_del17-60 to homo-oligomerize. Interaction studies were performed using **A)** YFP-based bimolecular fluorescence protein complementation (YFP-BiFc) and **B)** sandwich ELISA. The human MC3R/GHSR heterodimer was used as a positive control (pos.ctrl. [46]) and the transfection of a single construct as a negative control (neg.ctrl.). Data was assessed from a minimum of three independent experiments. All measured interaction were expressed as % fluorescent cells to positive control (A) or as % absorption (492 nm/ 620 nm) relative to the positive control (B). Values represent mean + SEM. Significance was determined compared with the neg.ctrl. using one-way ANOVA and Dunnett´s test. In B, also significance between hGPR83 homodimer and hGPR83-del17-60 was determined (blue). ***P ≤ 0.001 (unpaired t-test, two-tailed).

Homo-oligomer formation of hGPR83 was detected by using two independent methods (Fig 5). The hGPR83 del17-60 variant, which exhibits constitutive activity (Fig 2), also forms oligomers according to the data received from bimolecular fluorescence complementation (BiFC; Fig 5A), but the capacity was lowered compared with wt according to results of a sandwich ELISA approach (Fig 5B). This method includes recognition of specific tags at the N- and the C-terminus and therefore the N-terminal tag, which must be recognized by a specific antibody for the detection of an interaction, may be sterically “invisible” in the extremely shortened hGPR83_del17-60 construct. We speculate that the used tag should be covered by the extracellular loops and can therefore not be recognized by the antibodies, which explains the decreased oligomerization detected using this method.

**Fig 6:**
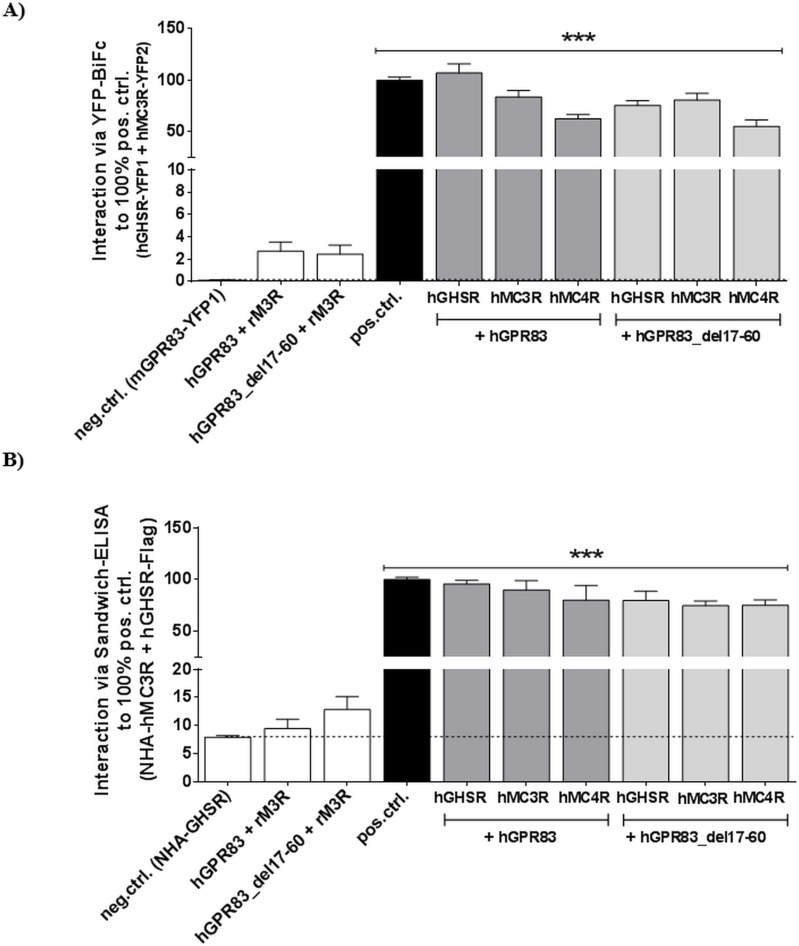
Capacity of wt hGPR83 and the eNDo deletion variant to interact with hGHSR, hMC3R, and hMC4R. Two methods were used to examine the capacity for oligomerization: **A)** YFP-based bimolecular fluorescence protein complementation (YFP-BiFc) and B) sandwich ELISA. For the sandwich ELISA, Flag-tagged hGPR83 constructs and NHA-tagged human GHSR/MC3R/MC4R respectively rM3R were used. The human MC3R/GHSR heterodimer was used as a positive control (pos.ctrl.) and the transfection of a single construct as a negative control (neg.ctrl.). All measured interactions were expressed as % fluorescent cells to the positive control (A) or as % absorption (492 nm/ 620 nm) relative to the positive control (B). Significance was determined compared with the neg.ctrl. using one-way ANOVA and Dunnett´s test. ***P ≤ 0.001.

However, hetero-oligomerization between wt hGPR83 or the hGPR83_del17-60 variant and the three hypothalamic GPCRs MC3R, MC4R, and GHSR, respectively, was clearly observed (Fig 6). In addition to heterodimerization, rM3R was found to non-interact with hGPR83 and hGPR83_del17-60 (Fig 6). Of note, no remarkable differences were found between the wt and deletion hGPR83 variant in the capacity for hetero-oligomerization, which excludes that the ectodomain significantly influences hetero-oligomerization and that increased basal activity modifies the capacity for oligomerization.

### Basal signaling properties of hetero-oligomers between hGPR83 and different hypothalamic GPCRs

**Fig 7:**
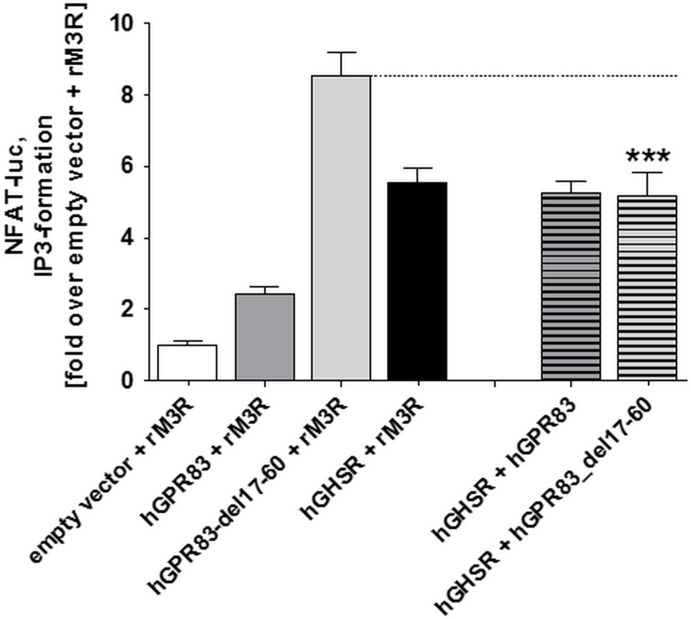
IP3 formation during co-expression of hGPR83 constructs with hGHSR in the basal state. Human GPR83 variants and hGHSR co-expressed with the non-interactive [5] cholinergic receptor, muscarinic 3 from rat (rM3R, Fig 6), served as assay controls and were compared with hGPR83 variant/GHSR interactions in the NFAT-driven signaling pathway. Data was assessed from a minimum of three independent experiments, each performed in triplicate, and calculated to the empty vector + rM3R set to 1. Values represent mean + SEM.

Basal capacities of hGPR83, hGPR83_del17-60, and GHSR for IP formation were examined as individual receptor populations or under co-expressed conditions, respectively (Fig 7). Of note, both wt hGPR83 and GHSR showed a remarkable high basal signaling capacity, whereby the basal activity of GHSR was higher than observed for hGPR83. Furthermore, the hGPR83_del17-60 deletion variant was found to have higher signaling capacities than wt hGPR83 or GHSR (Fig 7).

The human hGPR83 co-expressed with the hGHSR showed similar basal signaling activity as GHSR alone. Interestingly, co-expression of the hGPR83_del17-60 variant, which exhibits a 4-fold higher basal signaling compared with wt hGPR83, shows basal signaling as observed for wt hGHSR basal signaling (Fig 7). This points towards a dominant impact of the GHSR on basal signaling properties under co-expressed conditions with the human hGPR83.

**Fig 8:**
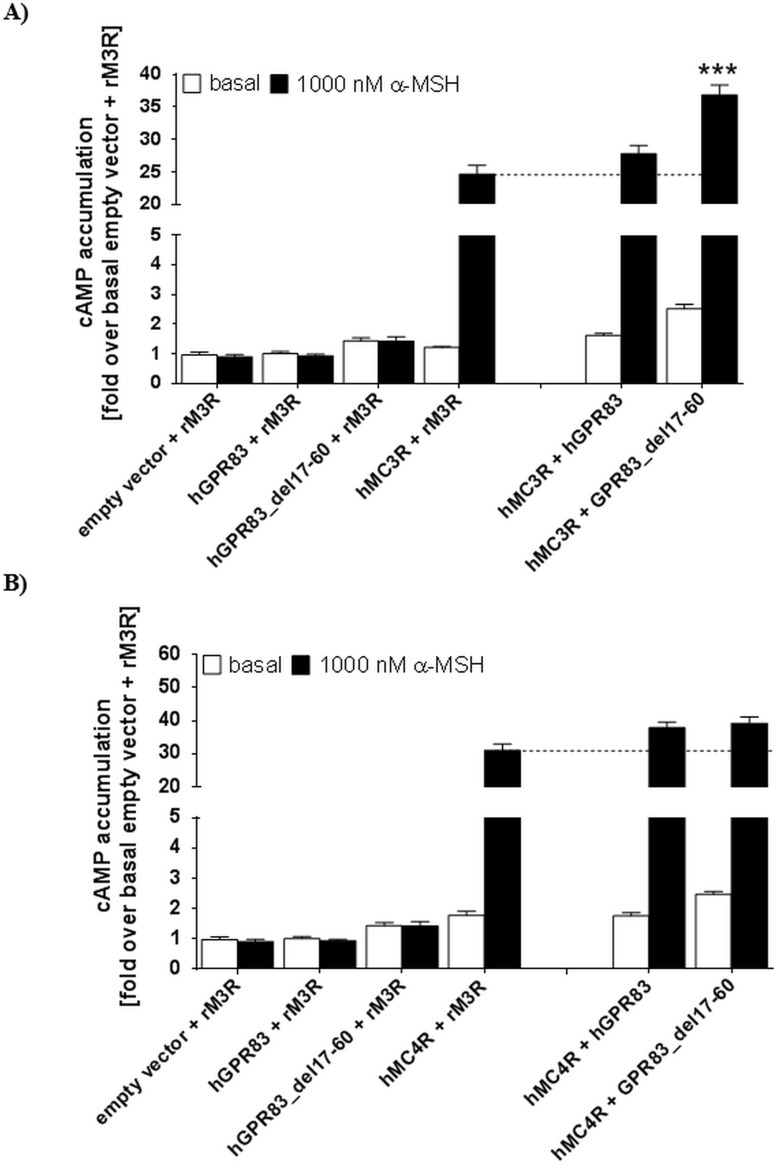
Gs-mediated signaling of MC3R and MC4R co-expressed with GPR83. We measured cAMP accumulation in the basal state and after stimulation with α-MSH in co-expression studies of **A)** wild type (wt) hGPR83 and hGPR83 del17-60 with wt hMC3R, and **B)** wt hGPR83 and del17-60 hGPR83 with wt hMC4R. Co-expression with the non-interactive cholinergic receptor muscarinic 3 from rat (rM3R) served as controls [5, 47] (Fig 6) and were compared with GPR83 constructs co-expressed with MC3/4R. Data was assessed from a minimum of three independent experiments, each performed in triplicate, and calculated to the empty vector + rM3R set to 1. Values represent mean + SEM. Significance was determined compared with MC3R + rM3R (A) and MC4R + rM3R (B) using one-way ANOVA and Dunnett´s test. ***P ≤ 0.001.

Moreover, co-expression of the human hGPR83 deletion variant with the human hMC3R enhances the basal activity state for Gs-mediated signaling by approximately 200%. Furthermore, the maximum level was increased after ligand application (approximately 1.5-fold; Fig 8A). This effect was not observed for the co-expression of hMC3R and wt hGPR83. A slight increasing effect on basal cAMP formation was also observable by the co-expression of the hGPR83_del17-60 variant with hMC4R (Fig 8B).

### Structural homology models of hGPR83 in different activity states—complexes with different intracellular effectors

The purpose of designing structural homology models of hGPR83 was to receive molecular insights into putative structural properties related to signaling regulation. Such knowledge can be helpful in generating ideas on the mechanisms of mutational effects and to provide hints on potential oligomeric properties, for either homo- or hetero-oligomeric constellations. Finally, these models will guide more rationally current and future experimental approaches focused on the molecular regulation and modification of hGPR83. We generated several structural human hGPR83 homology models: *1*. in an inactive state; *2*. in the active state interacting either with arrestin or with G-protein; *3*. a putative hGPR83 homodimer constellation; and *4*. a putative GPR83-MC4R heterodimer or hetero-oligomer constellation.

The hGPR83 model dimensions in the transmembrane region constitutes (position numbers for hGPR83 are provided): TMH1 start position 68, ICL1 100–104, TMH2, ECL1 136–140, TMH3, ICL2 175–182, TMH4, ECL2 207–236, TMH5, ICL3 266–287, TMH6, ECL3 318–323, and TMH7 end 348.

The experimental data in this study generated four main results: *i*. a multiple basal activity capacity of hGPR83; *ii*. the capacity to constitute oligomeric states, which may impact signaling; *iii*. the oligomeric interface(s) are located in the transmembrane region; and *iv*. conserved amino acids are located in the extreme N-terminal region close to TMH1, which are important for signaling regulation. Therefore, we designed the aforementioned structural homology models and complexes to provide insights to the following questions: How should the identified signaling sensitive amino acids be involved in the constitution of the basal receptor conformation? How can the hGPR83 homo- and hetero-oligomers be arranged?

#### The putative localization and interactions of signaling determinants in the ectodomain

**Fig 9:**
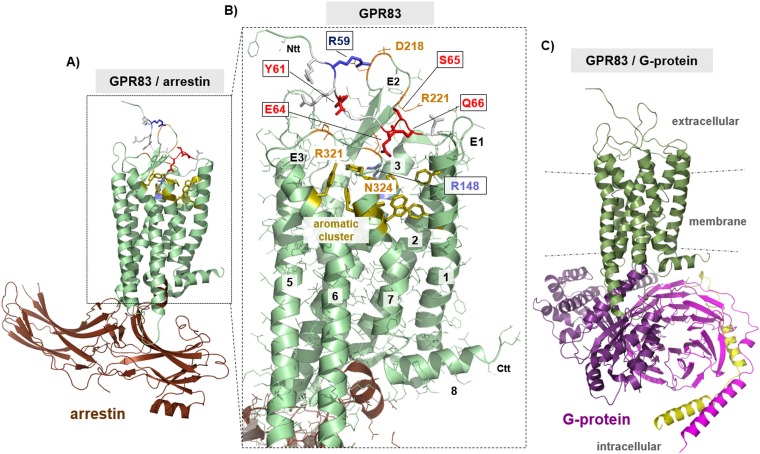
Structural homology models of hGPR83 in putative complexes with arrestin and G-protein. **A)** The presented structural hGPR83-arrestin complex homology model (backbone cartoon) is mainly based on the crystal structure of rhodopsin with bound arrestin (PDB entry 5DGY, [43]), which served as a template for modeling procedures. Several important aspects of putative hGPR83 components were additionally highlighted and visualized in detail in B). **B)** The constitutively activating mutants at positions 61–66 identified in this study are located at the transition between the eNDo and transmembrane helix 1. Alanine substitutions lead to, e.g., increased Gi, MAPK basal signaling activity, or IP formation. These amino acids are part of an extracellular fragment (amino acid side chains as sticks), which is embedded between the extracellular loops (ECL1-3 = E1–3 in Figure). Several hydrophilic amino acids (orange, lines), such as R321 (extracellular loop three (E3)) or R221 (E2), are in close spatial proximity and may be direct interaction partners. One suggested key interaction is a salt-bridge between E64 and R148 (in helix 3). Finally, arginine 59, a signaling sensitive residue characterized by a decrease in basal activity due to the alanine mutation, is suggested by this model to interact with negatively charged residues located in the extracellular loop 2 (such as with D218). Of note, closely below to these supposed hydrophilic interactions, a cluster of interacting aromatic residues can be observed, specifically between helices 1-2-6-7. **C)** The hGPR83 has been shown in several studies to interact with different G-proteins, such as Gq or Gi, also in the basal state [10, 48]. Such putative complex can be simulated by using the solved crystal structure of the ADRB2/Gs complex as a template (PDB entry 3SN6, [28]). The G-protein is a trimer, constituted by non-covalently bound subunits (alpha, lilac; beta, magenta; gamma, yellow). The diverse hGPR83 conformations (A and C) are characterized by specific differences to one another and particularly compared with the inactive conformation (Fig 10). E1–3, extracellular loops 1–3, Ctt, C-terminal tail, Ntt, N-terminus.

**Fig 10:**
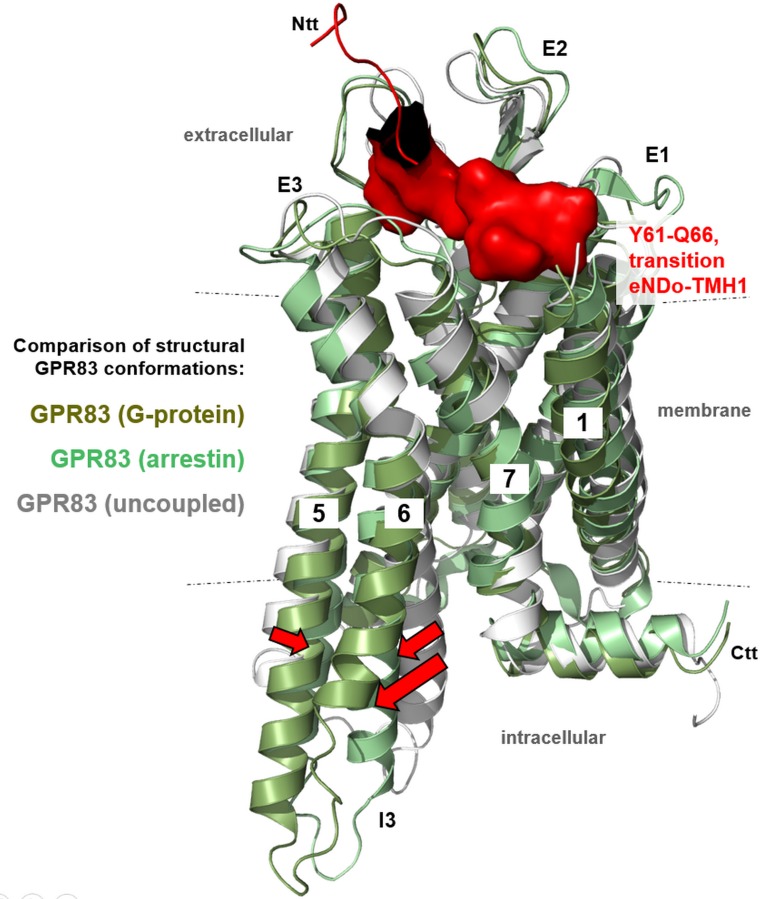
Comparison of different putative hGPR83 conformations in dependency of the signaling activity state and interaction partner. The homology models of hGPR83 either in interaction with arrestin or G-protein were superimposed with the model of an inactive state conformation (backbone cartoon representation, differentiated by colors and without an interaction partner). Furthermore, the three amino acids at the transition between the eNDo and transmembrane helix 1, which are determinants of basal signaling regulation, are indicated in red and are visualized by a partial surface. This illumination shows the close package of this part between the extracellular loops (E1–3). The entire eNDo is not included in these models, because a structural template or crystal structure of this part is not yet available. Activation of the receptor, induced by mutation or by ligand binding, is characterized by helical movements relative to one another, with the highest displacement of TMH6. This is also reflected by comparison of the three structural hGPR83 models (magenta arrows). Transmembrane helices 5 and 6 move during receptor activation to enable binding of the intracellular interaction partner (Fig 9). E1–3, extracellular loops 1–3, Ctt, C-terminal tail; Ntt, N-terminus; I, intracellular loop 3.

The amino acids at the transition between the eNDo and TMH1, which are determinants of basal signaling regulation (Fig 4), namely Y61/E64/S65 and Q66, are closely embedded between the extracellular loops according to our homology models (Fig 9 and Fig 10). Alanine substitutions of these residues lead to increased basal signaling activity and several hydrophilic amino acids, such as N324, R321 (ECL3), or R221 (ECL2), are in close spatial proximity to the wild-type residues. Our model suggests detailed interactions between E64–R148 or R321, S65–R221, and Q66–N324. Moreover, Y61 is in a central position between the N-terminus and the ECL2 and does interact via aromatic interactions with W236 (TMH5) and F228 (ECL2). One potential key interaction is a salt-bridge between E64 and R148 (helix 3). This interaction would anchor the extreme N-terminal ectodomain to the transmembrane domain. However, the alanine mutants of E64, S65, or Q66 may interrupt these suggested essential intra-molecular interactions, stabilizing the basal conformation, and leading to an increase in basal signaling by a “release” of the basal conformation. Of note, alanine mutants are often found to induce minor effects on signaling compared with more drastic changes in biophysical properties at a particular position by, e.g., substitutions to amino acids with completely opposite side chain properties, such as from aspartate (negatively charged) to arginine (positively charged). We therefore most likely registered only a slight increase in basal activity, which should be enhanced by other substitutions. However, the alanine mutations definitively point to amino acids of significance for activity state regulation (Fig 4).

Furthermore, arginine 59, a signaling sensitive residue suggested by a decrease in basal activity due to the alanine mutation (Fig 4), is proposed by this model to interact with negatively charged residues located in the extracellular loop 2 (E217 or D218). Charged interactions of functional importance at the extracellular receptor components are also known from other family A GPCRs, such as the ADRB2 [49]. We therefore suggest that the GPR83 conformation and signaling capacity is influenced at the extracellular site by such hydrophilic interactions.

Activation of the receptor, induced by mutation, ligand binding or other signaling-inducers, is characterized by helical movements relative to one another, with the highest displacement of TMH6 [28, 50]. This becomes also visible by comparison of the three different structural hGPR83 models (Fig 10). Transmembrane helices 5 and 6 move during receptor activation to enable binding of the intracellular interaction partners, such as beta-arrestin [51] or G-protein (Fig 9). This activation process requires a direct structural linkage between extracellular and intracellular receptor parts, which is especially determined by amino acids with a predisposition for activity regulation such as the here suggested extracellular amino acids.

Moreover, the hGPR83 constitutes homo-oligomers (Fig 5). In the present study, the formation of hetero-oligomers of GPR83 with other GPCRs has been found not to be dependent on the extracellular receptor part. Here, we provide a putative arrangement of the hGPR83 dimer with a common GPCR dimer interface between helices 1-2-8 (S2 Fig) [52]. Such an interface may be also involved in the formation of heteromers between GPR83 and MC4R. The MC4R, which has been shown in this study for the first time to have the capacity to heterodimerize with hGPR83, is also known to constitute homodimeric constellations, most likely with a protomer-protomer interface between transmembrane helices 3 and 4 and the intracellular loop 2 [44–46]. It can be therefore suggested that the homodimeric hGPR83 and MC4R arrangements may also form hetero-oligomers (S2D Fig), whereby the homodimeric interfaces still exist, but the heteromer contact is asymmetric and different compared with the homomeric interfaces.

## Discussion

The ectodomain of GPR83 orthologues is highly conserved, particularly the 30 amino acids close to TMH1 (Fig 3) and it was found on murine GPR83 that this receptor part has a functional relevance concerning activity regulation [11]. The eNDo may function as an intra-molecular ligand, in this case as an inverse agonist. However, it was not known which amino acids are of specific importance to maintain the basal state related structural conformation. To answer this question would be of enormous importance for three reasons. First, intra-molecular peptidic ligands (tethered ligands) are known for several other GPCRs such as the TSHR, PAR’s, or adhesion GPCRs [12] and are key players in receptor regulation. Second, the basal activity of GPCR strongly determines the general GPCR signaling properties, also in a time- and cell-dependent context [13, 14, 16]. Finally, the localization of important receptor residues for ligand binding and signaling regulation (or with a dual functionality) would help to understand the structural organization of this GPCR, which enables, together with functional implications, focus on potential tools for pharmacological interventions, for inhibition or activation.

### Modification of amino acids in the N-terminal domain leads to multiple constitutive signaling: identification of key residues for basal state maintenance

Firstly, we report that the hGPR83 exhibits a high basal activity for IP3 formation (most likely mediated by Gq/11 activation) and the N-terminal deletion construct del17-60 shows a 4-fold increased IP3 formation as the wild-type (Fig 2). This strongly supports similarities between the human (Fig 2) and murine GPR83 [11], which is also reflected by the fact that both mPEN and hPEN activate the hGPR83 [9]. We also describe activation of MAPK (arrestin activation) and G12/13 (RhoA) by deletion of the hGPR83 eNDo. This receptor is also characterized by a basal activity for Gi-mediated activity, whereby the eNDo deletion does not increase constitutive PLC activity compared with wt. We interpreted our data as a strong indication that hGPR83 exposes multiple basal signaling activation, which corresponds to the finding that PEN also activates several pathways in GPR83 expressing tissue and transient cell systems [9]. It must be noted that such multiple basal signaling activity is not evident for other GPCRs with high basal activity such as the thyrotropin (TSHR) or the GHSR. The TSHR also has the capacity to activate multiple signaling pathways after ligand (TSH) binding [53–58], but basal activity of wt TSHR is restricted to the Gs pathway. Our finding supports that the GPR83 is not selective for a specific intracellular effector, whereby the cellular context (cell type, G-protein availability) may be a determining factor of the predominantly active pathway. Moreover, deletion of the entire hGPR83 eNDo causes an increase in activity, which supports the previous notion [11] that the eNDo must interact with other receptor parts from the membrane spanning serpentine domain (SeDo, constituted by the 7 alpha-helices connected by loops), such as the extracellular loops (ECLs).

To identify such important amino acids, we performed site-directed mutagenesis of several residues which are, in consequence of the linear sequence (Fig 3) and in accordance to our homology models (Fig 9), most likely in close spatial proximity to the ECLs. The residues at the transition between the eNDo and the TMH1 may be predestined to be part of an intra-molecular ligand, which interacts with side chains of the loops or loop-helix transitions. We analyzed seven single side chains by alanine mutation and one triple mutant of three consecutive arginines to estimate their impact on basal signaling. Indeed, IP3 formation, MAPK and the G12/13 pathways were increased by mutations E64A, S65A, and Q66A. In contrast R59A, R60A, and the triple arginine mutations R58A/R59A/R60A slightly decreased these basal signaling pathways to various extents (Fig 4). Of note, the Y61A mutant decreased cAMP accumulation of forskolin-stimulated cells to approximately 50%, which indicates constitutive Gi/o activation. According to our homology models, this residue is tightly embedded between the eNDo, the ECL2, and linked transitions to helices and can interact with aromatic side chains located in TMH5 and the ECL2 (Fig 9). The designed homology models also suggest concrete interactions between the here identified signaling sensitive amino acids of the eNDo and diverse parts of the SeDo. Proposed interactions are (eNDo/SeDo) S65/R221 (ECL2), E64/R148 (TMH3), and Q66/N324 (ECL3). In turn, this region of the ectodomain is tightly embedded between other extracellular parts of the receptor and may function as an internal regulator or switch for activity-related conformational changes (Fig 10). By this, an interrelation between the extracellular parts and the intracellular region should be organized (allosteric coupling [59]). Altogether, these results point to single mutations, which are biased on their signaling impact. Mutation Y61A shows pronounced activation of Gi/o-mediated signaling. Other mutants either increase (such as Gq-mediated) or decrease basal signaling. This differentiation of the impact on different pathways provokes the assumption that particular receptor determinants at the extracellular part are in spatial relation to a differentiated signaling profile.

### hGPR83 homo- and heteromerizes by interactions in the transmembrane region

We also analyzed whether the hGPR83 was capable of constituting hetero-oligomers with other hypothalamic GPCRs. Of further interest was the impact of the eNDo on this capacity and how basal signaling is influenced by oligomerization. Firstly, the human receptor is capable of constituting homo-oligomers, also the truncated receptor variants with one another (Fig 5). As a consequence, under each circumstance, homo-oligomerization has to be considered by studying the mechanisms and properties of GPR83. Secondly, the hGPR83 can interact in heteromeric constellations with the GHSR, the MC3R, and also with the MC4R. These interactions, according to our set-up and data, cannot be disrupted by deletion of the eNDo (Fig 6), which strongly favors intermolecular contacts within the SeDo (S2 Fig).

For murine GPR83 a variety of heteromeric GPCR partners have already been identified, including the MC3R, GHSR [5], and the GPR171 [9]. Moreover, for co-expressed GHSR or GPR171 with GPR83, a mutual impact on signaling has already been confirmed [5, 9]. Such mutual interrelations provide the option to synchronize or to justify different ligand effects and physiological parameters related to the particular interacting GPCRs [60, 61].

With respect to the basal signaling capacity, we found the GHSR basal activity (IP3 formation, Gq activation), which is higher than that of wt hGPR83 but lower than the truncated hGPR83_del17-60 variant, to have a dominant impact on the overall outcome of ligand-independent signaling, as already observed for the mGPR83 in interaction with the GHSR [5]. Co-expressed receptors always demonstrate the basal signaling level of GHSR (Fig 7). This provokes the assumption that the GHSR, in a hetero-oligomeric state with GPR83, has a dominant basal signaling function. We also detected a slight increase in MC3R basal and maximum signaling (cAMP accumulation) when co-expressed with the truncated hGPR83 variant. The basal signaling of MC4R co-expressed with this GPR83 mutant is also increased in tendency (Fig 8). Finally, such receptor-receptor interactions must be considered as a determinant for basal signaling activity regulation, also *in vivo* if these receptors do occur simultaneously. This also highlights again for GPR83 the significance of heteromeric constellations on signaling properties, which also provides the option that modification of the oligomeric state should be a tool for the specification of directed interventions [52, 62]. On the other hand, it must be considered that directed pharmacological intervention at one GPCR will also modulate the function of the interaction partner, which may cause undesired side effects [63, 64].

## Conclusions and Open Questions

The data here provides support that hGPR83 can constitute homo- and hetero-oligomers with several different GPCRs, which has an impact on resulting basal signaling properties. Moreover, particular residues in the ectodomain may function as hubs for signaling regulation, either in the basal state or also in the ligand-induced state. We were able to show that the ectodomain of hGPR83 is of significant importance for a specified basal signaling regulation. These residues (and most likely others) may function as a tethered ligand, whereby the exact function during ligand action should be unraveled in future studies. Finally, we demonstrated that the basal activity and specific determinants located in the ectodomain contribute to signaling regulation.

## Supporting Information

S1 Fig Functional characterization of single side-chain variations in the N-terminus of hGPR83. Cell surface expression levels of different hGPR83 single amino acid substitutions compared to hGPR83 wild type. mGPR83 set to 100% served as positive control. As a negative control, untagged wt GPR83 was used. Mutants were compared to hGPR83 wild type using one-way ANOVA.
(TIF)

S2 Fig Homology models of putative oligomeric hGPR83 packing constellations. **A)** The hMC4R is known to constitute homodimeric constellations, most likely with a protomer-protomer interface between transmembrane helices 3 and 4 and the intracellular loop 2 [44–46]. Such constellations can also be found for crystal structures of the CXCR4 dimer, which served as a structural template for the MC4R protomer arrangement. B) The hGPR83 also constitutes homo-oligomers (Fig 5). For GPCRs, different potential dimeric or oligomeric interfaces between the interacting protomers are generally suggested based on biophysical studies or GPCR crystal structures (see material and methods, modeling section). We here show a putative arrangement of the hGPR83 dimer with a common GPCR interface between helices 1-2-8. The dimeric formation has been found in this current study not to be dependent on the extracellular receptor part, which corresponds with interactions between transmembrane receptor parts as shown in this dimer model. C) Such an interface may be also involved in the formation of heteromers between hGPR83 and MC4R. However, it can be suggested that the homodimeric hGPR83 and MC4R arrangements may also form hetero-oligomers as presented in D), whereby the homodimeric interfaces still exist, but the heteromer contact differs. For GPR83, a variety of heteromeric GPCR partners have been identified so far, including the MC3R, MC4R, GHSR, and the GPR171 [9]. E1–3, extracellular loops 1–3; Ctt, C-terminal tail; Ntt, N-terminus; I3: intracellular loop 3.
(TIF)

## Abbreviations

GPCR: G-protein coupled receptor
GPR83: G-protein coupled receptor 83
TSHR: thyrotropin receptor
ICL: intracellular loop
ECL: extracellular loop
TMH: transmembrane helix
Ntt: N-terminal tail
eNDo: extracellular N-terminal domain
SeDo: serpentine domain
MC3R: melanocortin-3 receptor
MC4R: melanocortin-4 receptor
GHSR: ghrelin receptor
CAM: constitutively activating mutation
PAR: protease-activated receptor
