# Supplementary figures and images for: Insights into Basal Signaling Regulation, Oligomerization, and Structural Organization of the Human G-Protein Coupled Receptor 83
Source: PLoS One. 2016 Dec 9;11(12):e0168260. doi: 10.1371/journal.pone.0168260 (PMC5148169; doi:10.1371/journal.pone.0168260)

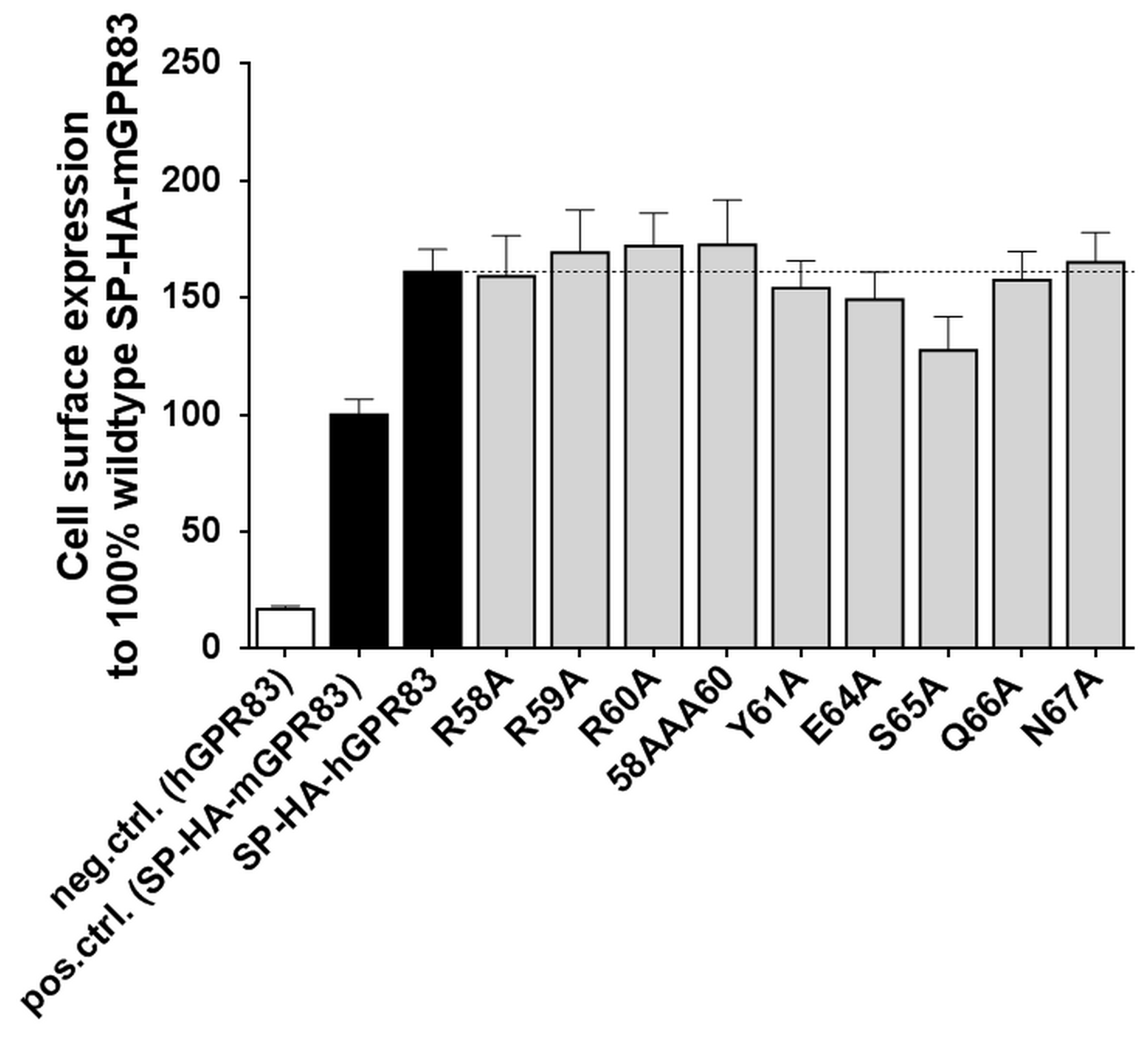

Supplement: S1 Fig — Cell surface expression levels of different hGPR83 single amino acid substitutions compared to hGPR83 wild type. mGPR83 set to 100% served as positive control. As a negative control, untagged wt GPR83 was used. Mutants were compared to hGPR83 wild type using one-way ANOVA. (TIF) [file pone.0168260.s001.tif]

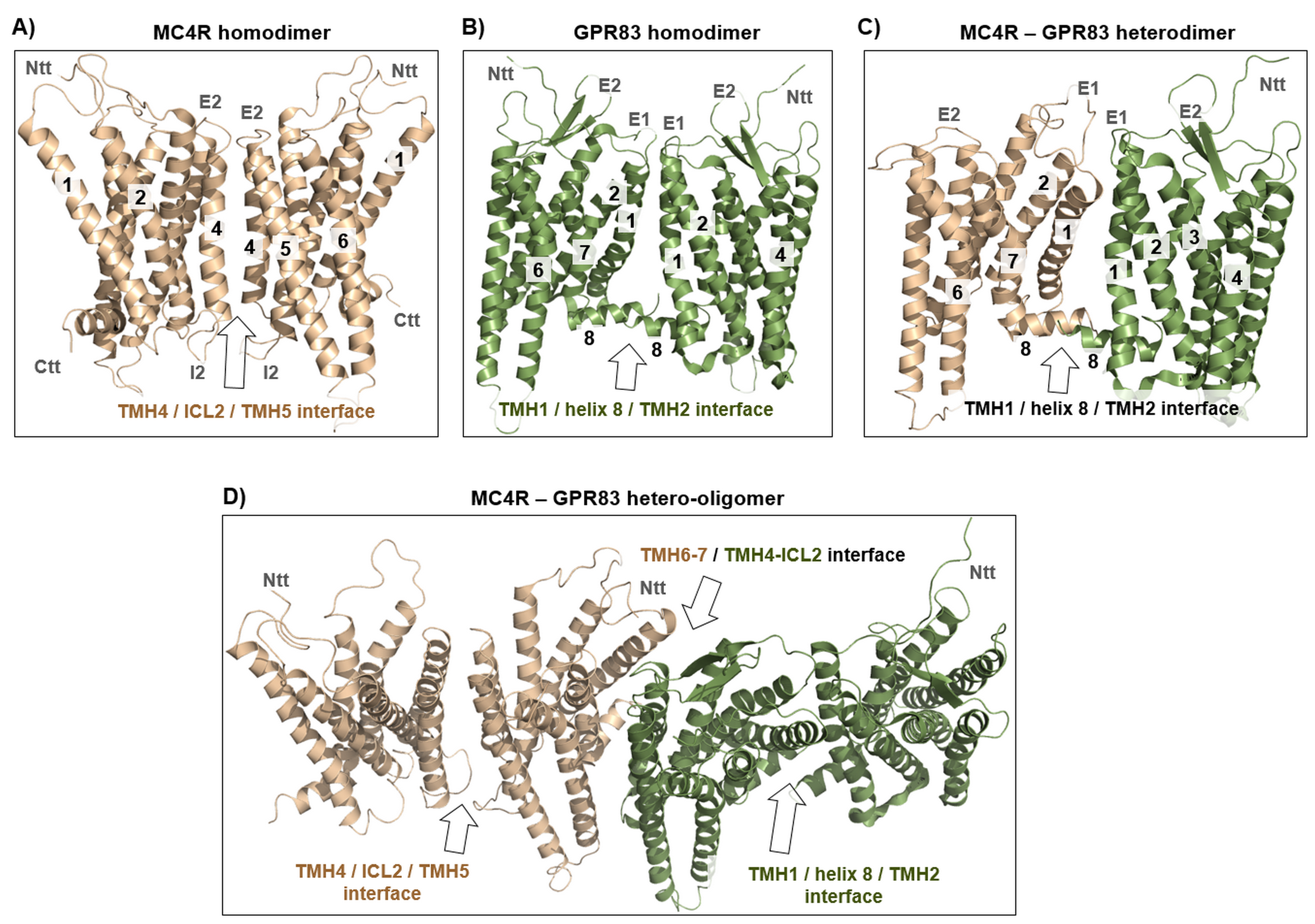

Supplement: S2 Fig — A) The hMC4R is known to constitute homodimeric constellations, most likely with a protomer-protomer interface between transmembrane helices 3 and 4 and the intracellular loop 2 [44–46]. Such constellations can also be found for crystal structures of the CXCR4 dimer, which served as a structural template for the MC4R protomer arrangement. B) The hGPR83 also constitutes homo-oligomers (Fig 5). For GPCRs, different potential dimeric or oligomeric interfaces between the interacting protomers are generally suggested based on biophysical studies or GPCR crystal structures (see material and methods, modeling section). We here show a putative arrangement of the hGPR83 dimer with a common GPCR interface between helices 1-2-8. The dimeric formation has been found in this current study not to be dependent on the extracellular receptor part, which corresponds with interactions between transmembrane receptor parts as shown in this dimer model. C) Such an interface may be also involved in the formation of heteromers between hGPR83 and MC4R. However, it can be suggested that the homodimeric hGPR83 and MC4R arrangements may also form hetero-oligomers as presented in D), whereby the homodimeric interfaces still exist, but the heteromer contact differs. For GPR83, a variety of heteromeric GPCR partners have been identified so far, including the MC3R, MC4R, GHSR, and the GPR171 [9]. E1–3, extracellular loops 1–3; Ctt, C-terminal tail; Ntt, N-terminus; I3: intracellular loop 3. (TIF) [file pone.0168260.s002.tif]
